# Increased Risk Taking in Relation to Chronic Stress in Adults

**DOI:** 10.3389/fpsyg.2015.02036

**Published:** 2016-01-29

**Authors:** Smarandita Ceccato, Brigitte M. Kudielka, Christiane Schwieren

**Affiliations:** ^1^Alfred Weber Institute of Economics, Ruprecht-Karls-Universität HeidelbergHeidelberg, Germany; ^2^Department of Medical Psychology, Psychological Diagnostics and Research Methodology, Institute of Psychology, University of RegensburgRegensburg, Germany

**Keywords:** chronic stress, gender differences, risk, self-reported measures, hair cortisol, C91, D81, D87, J16, 2340, 2560, 3360

## Abstract

Chronic stress is a public health problem that affects a significant part of the population. While the physiological damage it causes is under ongoing scrutiny, its behavioral effects have been overlooked. This is one of the first studies to examine the relation between chronic stress and decision-making, using a standard lottery paradigm. We measured risk taking in the gain domain through binary choices between financially incentivized lotteries. We then measured self-reported chronic stress with the Trier Inventory for the Assessment of Chronic Stress (TICS). We additionally collected hair samples in a subsample of volunteers, in order to quantify accumulation of the stress hormone cortisol. We discovered a significant positive, though modest, correlation between self-reported chronic stress and risk taking that is stronger for women than for men. This confirms part of the findings in acute stress research that show a connection between higher stress and increased risk taking. However, unlike the biologically-based results from acute stress research, we did not identify a significant relation between hair cortisol and behavior. In line with previous literature, we found a clear gender difference in risk taking and self-reports: women generally take less risk and report slightly higher stress levels than men. We conclude that perceived chronic stress can impact behavior in risky situations.

## Introduction

Understanding behavior under stress has received considerable research attention recently. The reason for this is the observation that stressors seem to have multiplied proportionally with the amount of political and economic uncertainty, and more and more individuals are affected by it (Anderson et al., 2010). Stress as a physiological phenomenon is double-sided: it has initially evolved as a useful, acute response to threat or challenge that marshals metabolic resources to adapt to short-term survival needs. However, when prolonged or having multiple sources, stress fosters chronicity which leads to disease and negatively affects bodily systems, including those involved in cognition and decision-making (McEwen and Sapolsky, 1995; Lupien and McEwen, 1997; Lupien and Lepage, 2001; Juster et al., 2010). Despite its importance, the knowledge of how exactly chronic stress affects cognitive mechanisms, decisions, and thus behavior, remains limited, as most of the existing research deals with acute stress. But, if chronic stress significantly alters cognition and decision-making processes, it is especially important to uncover to which extent and in what manner this happens.

A well-studied and important category of decisions are decisions under risk. Acute stress research has used financial risk taking paradigms to study decision-making under stress. Even though results are heterogeneous in terms of direction, the current conclusion is that risky decision-making under acute stress is altered (Starcke and Brand, 2012). This finding is not only relevant for overstressed stock traders that influence entire economies, but also for other groups at risk as, for instance, public employees dealing with emergency situations—like firemen, medical doctors, or policemen (Trautmann, 2014). It has also been shown that the cortisol levels of traders increase with increasing contextual uncertainty (Coates and Herbert, 2008), i.e., there might even be feedback effects. Thus, with increasing levels of stress, prolonged exposure to stress and the multiplication of stressors, not only risk taking behavior under acute but also under chronic stress becomes an important issue. We therefore propose one of the first investigations of decision-making under chronic stress^1^ and accumulated cortisol exposure and aim to assess if the reported effects of momentary stress on decisions maintain, especially those on risk taking behavior.

## Existing literature

Stress has been defined as the specific physiological response that the body initiates when confronted with an unpredictable or uncontrollable demand, i.e., a threat or a challenge, which triggers changes in homeostasis (Koolhaas et al., 2011). This acute, momentary response comprises the activation of the hypothalamic-pituitary-adrenal (HPA) axis, assessable through cortisol release, and the activation of the sympathetic-adrenal-medullary (SAM) axis, assessable through adrenaline release (Dickerson and Kemeny, 2004; van Stegeren et al., 2008). If repeated or prolonged, stress becomes chronic and the bodily systems become exhausted, paving the way for disease (McEwen, 2004). Up to date, there is no single measure for chronic stress as robust and valid as salivary cortisol for acute stress, although several biological measures have been scrutinized (McEwen, 2000; Wüst et al., 2000) and some optimistic perspectives have been offered (Klein et al., 2004; Stalder and Kirschbaum, 2012a). However, for both acute and chronic stress, there is another measurable facet in humans: the perceived experience of stress. To-date, several valid self-report measures have been developed for chronic stress (Cohen et al., 1983; Levenstein et al., 1993; Schulz et al., 2004) and, in acute stress research, visual analog scales and momentary emotional assessments are often utilized (for instance, see Kirschbaum et al., 1999).

Given these two different approaches to capture stress, it is of importance to elucidate the correlation between physiological measures of stress and self-reported experience. Cortisol release fluctuates with induced changes in affect (Buchanan et al., 1999), but the relation between biologically measured and self-reported acute stress is characterized by heterogeneous results (for associations see Hellhammer and Schubert, 2012; for a time-dependent relation Vinkers et al., 2013; for the lack of association Buchanan et al., 1999). However, recently it could be shown that acute endocrine responses lag behind acute psychological responses but that time-lagged correlations lead to a much closer coupling between verbal and physiological responses than reported in earlier studies (Schlotz et al., 2008). For the relation between self-reported chronic stress and biological measures of chronic stress the jury is still out, since there is no gold-standard for biologically quantifying chronic stress. However, lately, more and more research in the field has concentrated on the novel, promising measurement of cortisol in hair.

Hair cortisol concentration (HCC) is an intraindividually stable measure of chronic HPA axis activity that can provide a retrospective calendar of accumulated cortisol exposure^2^. Therefore, HCC has been introduced as a promising measure to capture chronic stress biologically (Meyer and Novak, 2012; Russell et al., 2012; Stalder and Kirschbaum, 2012a; Stalder et al., 2012b). While possibly affected by hair-washing frequency (Hamel et al., 2011), it appears to be robust to hair-coloring or other typical confounders of cortisol measures like smoking (Dettenborn et al., 2012). Indeed, research has reliably reported increased HCC in, for example, stressed neonates, shift workers, unemployed individuals, high-endurance athletes, individuals suffering from chronic pain, having been through major life events, or having some mental disorders (Kirschbaum et al., 2009; D'Anna-Hernandez et al., 2011; Gerber et al., 2012; Staufenbiel et al., 2013). Also, HCC correlates with major life events, caregiving burden (Stalder et al., 2014) and perceived stress (Karlén et al., 2011). In particular, there exists one further study that reports on an association between HCC and subjective stress as measured by the social overload scale of the Trier Inventory for the Assessment of Chronic Stress (TICS) (Stalder et al., 2012c). There also exist some reports on associations between perceived chronic stress and HCC at least in special subpopulations like unemployed individuals (Dettenborn et al., 2010), pregnant women (Kalra et al., 2007) or in a subsample of the general population (O'Brien et al., 2013). However, HCC seems not to be generally correlated with perceived chronic stress (Dowlati et al., 2010; Karlén et al., 2011; van Holland et al., 2012). Notwithstanding, it is of importance to acknowledge that potential associations may not maintain ad infinitum a positive trend, as an initial high cortisol reactivity (and correlated HCC levels), when prolonged, might exhaust the physiological systems and later result in hypoactivity (Kudielka et al., 2006). In fact, a recent report on hair cortisol, perceived stress and health shows that this might be the case. Greek youth, who have been subject to multiple, prolonged acute stressors owing to major national economic difficulties, report higher perceived stress, more depressive symptoms, anxiety, and major life events while having lower hair cortisol levels than equivalent Swedes (Faresjö et al., 2013).

It is not only the development of biological markers for chronic stress and the establishment of hyper vs. hypocortisolemic patterns under chronic stress that received less attention than acute stress in the field, but also basic research concerning how chronic stress affects cognition, decision-making, and thus behavior. In terms of altered cognition, it has repeatedly been shown that acute stress negatively affects reaction times, feedback learning and learning from negative outcomes. It further has negative effects on memory (Preston et al., 2007; Buchanan and Tranel, 2008; Smeets et al., 2008; Petzold et al., 2010). Attention tunneling might actually have beneficial effects, facilitating the disregard of peripheral information (Staal, 2004). Yet, for chronic stress, the way it affects cognitive mechanisms is less researched experimentally and current conclusions are based on limited evidence.

Initial proposals assert that chronic stress generally affects performance in individuals with high stress sensitivity (Baradell and Klein, 1993) and adversely impacts neurological structures involved in learning, memory and decision-making (Lupien and McEwen, 1997; Lupien and Lepage, 2001), disrupting, among others, excitatory working memory networks (Hains et al., 2009). While it seems to have no effect on reaction times (Schwabe et al., 2008), chronic stress appears to affect learning and memory similarly to acute stress (Schwabe et al., 2010), targeting the quality of learning: stimulus-response learning strategies, i.e., habit learning, are used instead of more flexible strategies (Schwabe et al., 2008). Also, short-term memory processing is slower (Brand et al., 2000) and long-term memory is probably affected because of specific decrease of gray matter volume in the hippocampus (Gianaros et al., 2007). Finally, a process that might be promoted is the memory for fear-arousing events. Chronic stress seems to improve it and thus lead to a higher sensitivity for negative experiences, a propensity to potentially see risks and threats where none exist, and to experience depression and learnt helplessness (Korte, 2001; Luethi et al., 2008).

The only existing study on chronic cortisol exposure and decision-making showed that exogenous cortisol administration increased risk aversion (Kandasamy et al., 2014) while studies on decision-making under acute stress report increased risk seeking behavior (see below). In that study, Kandasamy et al. (2014) administered hydrocortisone (pharmaceutical cortisol) in a placebo-controlled, double-blind study to 36 participants of both genders over 8 days. They then asked participants to complete several tasks assessing risk preferences and discovered that while the acute cortisol increase has no effect on risk aversion, chronic cortisol exposure increased risk aversion independently of gender. However, we would hesitate to equate chronic cortisol exposure to chronic stress^3^. Regarding biologically assessed acute stress, evidence showed that decision-making under uncertainty is significantly affected (Starcke and Brand, 2012; Buckert et al., 2014). Possibly in a time-dependent manner (Pabst et al., 2013c), risk taking increases under acute stress (Preston et al., 2007; Starcke et al., 2008; Lighthall et al., 2009; van den Bos et al., 2009; Pabst et al., 2013a). Though, there is also some counter-evidence (Lempert et al., 2012; Delaney et al., 2014; Gathmann et al., 2014). This heterogeneity in results is, as detailed in Buckert et al. (2014), probably due to the heterogeneity in stressors and design-relevant factors like the decision domains or the different ways of varying probabilities and reward values in the tasks. For instance, the decision-making domain seems to yield differential effects under stress. In particular, acute stress merely seems to increase risk taking in the gain domain. Others, however, report a stronger reflection effect (Porcelli and Delgado, 2009) or no effect (Pabst et al., 2013b).

Finally, acute stress seems to potentiate gender differences in risk attitudes. While most people are generally risk averse (Bernoulli, 1954; Coates and Herbert, 2008; Guiso et al., 2013), women are more risk averse than men (Eckel and Grossman, 2008). Under acute stress, this general difference has been found to be enhanced: risk aversion increases in women and risk seeking increases in men (Preston et al., 2007; Lighthall et al., 2009). However, other data could not confirm this effect (Starcke et al., 2008; Pabst et al., 2013a,b) or reported that this differential effect disappears if stress-related cortisol levels are very high (van den Bos et al., 2009).

Here, we set out to explore decision-making in relation to subjective perceived chronic stress and long-term cortisol exposure in uncertainty conditions using the standard risk taking paradigm employed in stress research and behavioral economics.

## Hypotheses

**Hypothesis 1** Despite mixed results in the literature, our own research (Buckert et al., 2014) shows that risk taking correlates positively with acute stress. Given that chronic stress presupposes prolonged or multiple exposures to acute stressors, we expect that also chronic stress is positively related to risk taking.

**Hypothesis 2** A recent study showed that chronic cortisol administration decreased risk taking, i.e., increased risk aversion. We expect that accumulated cortisol, as measured in hair samples, will also positively relate to risk aversion.

**Hypothesis 3** When assessing past experience and affect, women report higher levels of distress and specific symptoms than men. We expect to replicate this gender difference in the applied self-report chronic stress measure (TICS).

**Hypothesis 4** It has been reliably shown that men are more risk seeking than women. We expect to replicate this finding applying a standardized lottery paradigm.

## Experimental design

### The main risk task

**Figure 1:**
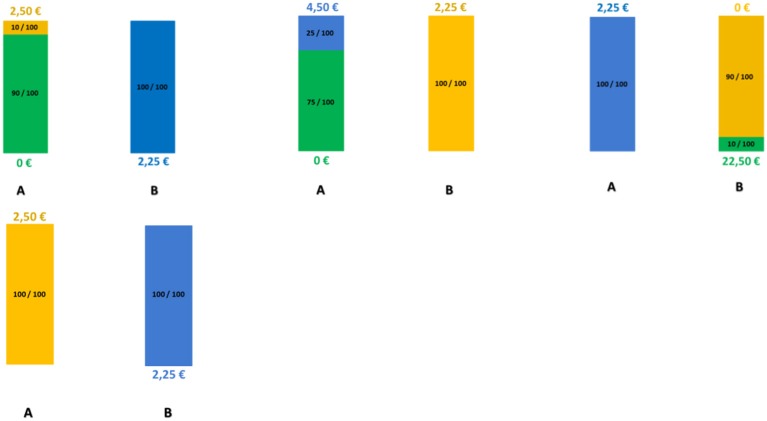
**Example of task trials**. Four sample pages from the task. **First row:** Examples of trials involving risky choices. Colors indicate the proportion of balls in the urn. Winnable amounts are written in the corresponding color and the proportion of balls is written on the urn, on each related colored segment. It is also indicated by the height of the colored segment. **Second row:** One example of a control trial.

We measured financial risk taking behavior in the gain domain in a pen-and-paper incentivized task, which we followed up with a spontaneous, real investment decision (more details are outlined in Procedure) and an item exploring self-reported general risk taking. The risk taking task followed, for comparability, a standard paradigm and consisted of 25 binary choices between a safe lottery^4^ offering 2.25 € and a risky lottery, a supplement of 5 binary choices between the same safe lottery and an ambiguous lottery^5^, as well as 3 control trials proposing choices between the safe lottery and another safe lottery offering a higher amount. The task follows the design used in Buckert et al. (2014). Each of the 33 choices was randomly displayed on a separate page. On each of the 33 task pages participants saw two options (Figure 1) and circled the one they chose.

The trials are depicted in Figure 1 and details regarding the options are presented in Table 1. The 25 trials involving risk combined five reward values, from 2.50 to 22.50 €, and five winning probability levels, from 10 to 90%, exploring a wide range of risky decisions (see Table 1). Given that the alternative option for these risky urns was always a safe urn offering 2.25 €, the combinations of value and probabilities yielded 10 urns where the expected value was smaller than what the safe one offered, five urns with equal expected value to that of the safe urn, and 10 urns with a higher expected value than that of the safe one.

**Table 1:** **Description of task trials**.

**Type of trial**	**Probability of receiving the non-zero amount (%)**	**Non-zero amount receivable upon choosing the alternative urn**				
		**2.50 € (+11.11%)**	**3.00 € (33.33%)**	**4.50 € (+100%)**	**9.00 € (+300%)**	**22.50 € (+900%)**
Risk	10	0.25 €	0.30 €	0.45 €	0.90 €	2.25 €
Risk	25	0.63 €	0.75 €	1.13 €	2.25 €	5.63 €
Risk	50	1.25 €	1.50 €	2.25 €	4.50 €	11.25 €
Risk	75	1.88 €	2.25 €	3.38 €	6.75 €	16.88 €
Risk	90	2.25 €	2.70 €	4.05 €	8.10 €	20.25 €
Control	100	2.50 €	3.00 €	4.50 €	–	–

The cells in columns 3–7 display the expected values of the 33 randomly played task trials. Choices are always made between a safe urn yielding 2.25 € and a risky, ambiguous or control urn. The first five rows (columns 3–7) display the expected values of the 25 risky urns, with expected values equal to the safe choice on the bottom-left to top-right diagonal. The last row (columns 3–7) presents the expected value of the three control urns.

All urns coded their specific uncertainty level through colored bar segments^6^ as in Buckert et al. (2014), and Putman et al. (2010), and they actually existed in the form of bags containing colored balls in various proportions (photos attached in Appendix in Supplementary Material). Participants were shown the bags with colored balls and were assured that the task is real. The potential reward of each urn was written, in the corresponding color, above and under the bar for lotteries containing two types of balls, and either above or under the bar for safe lotteries containing one type of balls only. A safe lottery was represented by an urn that contained 100 balls of the same color (Figure 1, choice B in the first pictogram). A draw from this urn would always result in the same colored ball, i.e., a blue ball, and would yield 2.25 €. A risky lottery, on the other hand, was represented by an urn which had two differently colored balls (Figure 1, choice A in the first pictogram). A draw from this urn would result in one of the two colored balls, i.e., either a yellow ball or a green ball, and would yield either 0 or 2.50 €. Finally, three control trials were introduced to make sure participants understood the task and are not providing automatic or naive responses by, for instance, choosing always urn A, indifferent of the reward they could get or the number of balls associated with a particular reward. A control trial consisted of a binary choice between two safe urns, i.e., urns containing 100 same-colored balls. A draw from any of these two urns would always yield the same payoff, since all balls had the same color (Figure 1, last pictogram). However, each of the two urns in the control trials offered a different reward: one always offered the 2.25 € while the other offered a higher amount (see Table 1, last row).

In all types of trials, all factors (color, positioning) were randomized. Also, there was no feedback for the decisions and no time limit for completing the task.

In order to analyze the data from this task, we quantified risk taking into a metric variable ranging from 0 to 25. This represents the frequency of choosing the risky urn in the 25 binary choices representing risk taking (rows 1–5 in Table 1). Task understanding was measured with a metric variable ranging from 0 to 3. It encodes the frequency of choosing the safe urn with the highest payment.

### Procedure

The experiment was conducted at the University of Heidelberg, Germany. Participants were recruited either during coursework and completed the experiment in class, or from our participant pool, using ORSEE (Greiner, 2004) and completed the experiment in the laboratory. In class, participants were seated in every other seat for privacy reasons while in the laboratory they were seated in private cubicles. Participants' information, responses and choices remained anonymous. The experiment started with reading the instructions aloud, so that participants would understand that everybody else was accomplishing the same task (instructions are attached in the Appendix in Supplementary Material). While describing the urns, the research assistants showed the actual bags containing the colored balls (photos in the Appendix in Supplementary Material) and explained that the payment of each participant will be decided, at the end, by herself/himself, through drawing a ball out of the corresponding bag. Further, participants were told that they would also determine, by drawing one code out of the 33 possible, which of the trials will be played, i.e., for which specific probability distribution they have to draw a ball from the corresponding bag. In this way, both randomness and trust in the correctness and fairness of the procedure were assured through transparency. Finally, participants were also introduced to the possibility of donating, at the end of the study, a hair sample.

Further, self-reported risk taking was measured through a visual analog scale ranging from 0 to 10, where participants pinpointed their general willingness to take risks. This measure has been previously validated as a good approximation of real-world behavior under risk (Dohmen et al., 2011).

After the participants completed the task and the demographic and psychometric questionnaires, they went to the payment table, one by one, and their final payoff (containing a 3 € show-up fee plus decision based pay) was determined.

Finally, after receiving the payment, the participant was asked to invest the amount he/she just gained, partly or in total, in a gamble that offered the chance of doubling the investment. This new lottery was constructed as a real-world risk taking task where money, one has invested effort for, is at stake. The outcome of the investment was decided by a coin flip. The participant himself/ herself threw the coin and had a 50% chance of doubling the invested amount and a 50% chance of losing it entirely. We measured this risk taking behavior with a metric variable showing the proportion of the experiment's payoff that the participant invested (from 0 to 100%).

### Participants

The total sample included 205 young adults who participated in the study either in class (*N* = 67) or in the laboratory (*N* = 128)^7^. The final sample used for analysis included 195 observations, as nine participants were excluded because of taking prescription medication for psychiatric conditions and one participant was excluded because of misunderstanding the lottery task. The mean age of our final sample was 22.74 years and ranged from 18 to 33 years with 57% women and 43% men. Fifty-one of the 195 participants additionally volunteered to donate hair for cortisol analysis. These participants did not differ significantly in any relevant respect from those who did not donate hair. This study was carried out in accordance with the recommendations of the guidelines of the American Psychological Association and approved by the Ethics Committee of the German Psychological Association. All subjects gave written informed consent in accordance with the Declaration of Helsinki.

### Control variables

We aimed to uncover the relationship between perceived chronic stress, accumulated cortisol in hair, and risk taking controlling for age, income, expertise in economics, acute stress experience, trait anxiety, depressiveness and general stress reactivity, medication for chronic disease and oral contraceptive use. To measure acute stress, we elicited momentary self-reports through a visual analog scale from 0 to 100 (as in Kirschbaum et al., 1999). To screen for depression and anxiety, we used the validated German version of the HADS, the Hospital Anxiety and Depression Scale (Herrmann-Lingen et al., 2011). Finally, we included, as a brief measure of stress reactivity, the short five-item version of the Perceived Stress Reactivity Scale (PSRS, Schlotz et al., 2011). Some other items were collected in a brief self-report form [e.g., age in years, available income in Euro, economics major (yes or no)].

### The chronic stress measure

Self-reported chronic stress across the last month was measured by the validated German TICS questionnaire (Schulz and Schlotz, 1999; Schulz et al., 2004). It is comprised of nine subscales (“excessive workload,” “excessive social demand,” “pressure to be successful,” “dissatisfaction at work,” “mental overload at work,” “lack of social recognition,” “social tensions,” “social isolation” and “chronic anxiety”) with a total of 57 items. The answering format follows a 5-point Likert scale (“never,” “infrequent,” “sometimes,” “frequent,” “and very frequent”). The TICS can be completed in 10–15 min.

We used naturally-occurring levels of chronic stress toward the end of the winter semester, in November to December 2012 and January to February 2013. This period includes handing in final reports and projects, exam preparation and exam taking. As the span of the stressful period is rather short, we modified the original TICS questionnaire and assessed experience pertaining to the last month instead of the last 3 months. The inter-item reliability analysis showed that our modification is valid, as the version we employed is as reliable as the original version^8^. To avoid multiple testing and maintain the standard we used in another study (Ceccato et al., in preparation), we followed the TICS scoring procedure from Schwabe et al. (2008). We summed up a total chronic stress score by adding the 57 items into a continuous variable.

### The hair cortisol measure

We collected hair samples from voluntary participants as described in Kirschbaum et al. (2009) and as instructed on the webpage of the Biopsychology Laboratory, Dresden University^9^, where samples were then analyzed. We used fine scissors to cut two hair strands from two sites in a posterior vertex position, as close as possible to the scalp. Since we modified the perceived chronic stress questionnaire to reflect the participant's experience in the latest month, we collected samples of minimum 1-cm segments closest to the scalp and we ordered analyses for this proximal segment by the biochemical laboratory of the University of Dresden, Germany (Prof. Kirschbaum). The average weight per hair segment was 7.5 ± 0.5 mg. A commercially available immunoassay with chemiluminescence detection (CLIA, IBL-Hamburg, Germany) was used to determine cortisol concentration from hair. The intraassay and interassay coefficients of variation of this assay are below 10%. We additionally collected relevant data in connection to the hair samples like number of hair washes per week, hair treatments, and natural hair color.

### Statistical analysis

We analyzed the data using SPSS version 20 with two-tailed tests for the undirected hypotheses and one-tailed tests for the directed hypotheses. The significance threshold was set at *p* < 0.05. Behavior was analyzed by performing appropriate correlations between variables denoting chronic stress or chronic cortisol exposure and risk taking and risky investment. To test the hypotheses that required mean comparisons we used either *t*-tests or Mann-Whitney U tests, in function of data normality. Finally, we assessed the robustness of the results in an OLS regression model controlling for all measured confounding variables.

## Results

### Association between perceived chronic stress and choices

We begin by testing Hypothesis 1 exploring whether perceived chronic stress, measured by the TICS, is associated with choices in uncertain contexts. Table 2 presents descriptive statistics for the main variables in our study and, as gender differences in risk taking have been frequently shown in the literature, the values are calculated for the overall sample as well as for each gender separately. Further, Table 3 includes the correlation coefficients and corresponding significance levels for tested associations. To assess behavior under uncertainty, we use the propensity to choose risky gambles in the task. We supplement our analysis with results from self-reported risk taking, and participants' investment in a real gamble. The propensity to choose risky gambles in the task is calculated as the frequency of choosing the risky lottery in the 25 risky trials. On average, participants chose 10.07 risky lotteries out of 25 possible. This is well aligned to expectations, as exactly 10 lotteries were offering a higher expected value than the 2.25 € safe alternative^10^. First of all, results in the total study sample show that we can accept Hypothesis 1, since there is a significant positive correlation between risk taking as measured by our incentive compatible task and the TICS sum score (*r* = 0.18^*^; *p* = 0.011).

**Table 2:** **Descriptive statistics for the main variables**.

**Variable**	**Overall**	**Women**	**Men**
*N*	195	84	111
Age—M	22.74 (2.46)	22.46 (2.07)	22.95 (2.71)
% Females	43.0%	100%	0%
HCC (pg/mg)−*N*	7.04 (3.61)	6.26 (3.75)	7.59 (3.45)
	51	21	30
TICS	83.50 (26.03)	87.43 (24.71)	80.52 (26.71)
Risk-taking frequency (task)	10.07 (3.47)	8.80 (3.30)	11.04 (3.29)
Risk-taking (self-report) −	5.17 (1.92)	4.79 (1.93)	5.45 (1.87)
Investment %	36.34% (39.21)	23.92% (30.90)	45.77% (42.26)

The table displays descriptive statistics for the main variables: mean, SD in parenthesis.

**Table 3:** **Correlations^11^**.

		**Stress variable**	**Coefficient (*r* or *r_*s*_*)**	**Overall *r*/*r_*s*_* (*p*) *N***	**Females *r*/*r_*s*_* (*p*) *N***	**Males *r*/*r_*s*_* (*p*) *N***
Behavioral variable	Risk-taking frequency (task)		*r*	0.18 (0.006) 195	0.29 (0.004) 84	0.20 (0.017) 111
	Risk-taking (self-report)	TICS	*r*	0.10 (0.094) 195	0.24 (0.014) 84	0.035 (0.360) 111
	Investment %		*r_*s*_*	0.01 (0.453) 183	−0.06 (0.312) 79	0.09 (0.189) 104
	Risk-taking frequency (task)		*r_*s*_*	0.15 (0.141) 51	0.18 (0.221) 21	−0.02 (0.461) 30
	Risk-taking (self-report)	HCC (pg/mg)	*r_*s*_*	0.14 (0.158) 51	0.07 (0.380) 21	0.04 (0.408) 30
	Investment %		*r_*s*_*	0.21 (0.081) 47	0.03 (0.452) 20	0.31 (0.060) 27
Stress variable	TICS	HCC (pg/mg)	*r_*s*_*	−0.16 (0.260) 51	−0.18 (0.437) 21	−0.14 (0.459) 30

The table displays appropriate correlation coefficients for either Pearson (r) or Spearman (r_s_) tests, as well as the sample size for each correlation analysis (second line, under the parentheses, denoted by N). Correlations significant at the 0.05 level are denoted by “^*^” and those significant at the 0.01 level by “^**^”.

Interestingly, the two other risk-taking measures lead to slightly different results: Participants' average general risk attitude as indicated in the visual analog scale was 5.17 (*SD* = 1.93), while in the behavioral response task (offer to double or lose gain in new lottery by flipping a coin) subjects took risks 40% of the time (the correlation between the two yields *r* = 0.35; *p* < 0.001). In the total sample, self-reported general risk taking is only by trend related to perceived chronic stress while women's self-reports on general risk taking correlate significantly positive with perceived chronic stress levels (*r*_*s*_ = 0.25; *p* = 0.024). In respect to the behavioral response task, the proportion invested correlates significantly with both lottery-task-measured risk taking and self-reported general risk taking (*r*_*s*_ = 0.25; *p* = 0.001; *r*_*s*_ = 0.22; *p* = 0.003), but is not significantly related to perceived chronic stress levels (*r*_*s*_ = 0.01; *p* = 0.905), neither for the overall sample nor for males and females separately.

### Association of accumulated cortisol in hair with risk taking and perceived chronic stress

We measured accumulated cortisol over time by analyzing cortisol concentrations in hair samples. A subgroup of 51 subjects (26%) agreed to donate hair samples (27% of males, 25% of females). Further information concerning the hair samples and cortisol concentrations is included in the Appendix in Supplementary Material. HCC is not significantly related to any of the variables measuring decision-making under risk (see Table 3), but shows a positive association at trend level with the investment in the gamble for men (*r*_*s*_ = 0.31; *p* = 0.060). We therefore cannot confirm Hypothesis 2. Further, there was no significant correlation between HCC and perceived chronic stress.

### Gender differences

In the literature, gender differences in stress-self-reports have been repeatedly outlined. At trend level, this is replicated in the present data. Women report, on average, a sum score of 87.43 (*SD* = 24.71) on the TICS, while men report almost 10% less, 80.52 (*SD* = 26.71). Mean levels are presented in Table 2 and mean comparisons in Table 4 (*p* = 0.066; Cohen's *d* = −0.27). If we look at the biological facet of stress, the opposite trend can be observed: men have higher average hair cortisol concentrations (7.59; *SD* = 3.45) than women (6.26; *SD* = 3.75). However, this distinction is significant at trend level only (*p* = 0.075; Cohen's *d* = 0.370).

**Table 4:** **Mean comparisons between genders**.

	**Tested variable**				
	**TICS**	**Risk-taking frequency (task)**	**Risk-taking (self-report)**	**Investment %**	**HCC (pg/mg)**
*p*-value	0.066	<0.001	0.025	0.001	0.075
Test	*t*-test	*t*-test	MW	MW	MW
*N* (*N_*f*_*; *N_*m*_*)	195 (84; 111)	195 (84; 111)	195 (84; 111)	183 (79; 104)	51 (21; 30)

The table displays, for each tested variable, p-values, the applied test and the sample size for mean comparisons between gender subsamples.

Further, a significant gender difference emerged in self-reported general risk taking, where women situate themselves, on average, at 4.79 (*SD* = 1.93) on a scale from 0 to 10, while men at 5.45 (*SD* = 1.87). This difference (*p* = 0.025; Cohen's *d* = 0.347) predicts actual behavior under uncertainty (see below).

In our data, women reported to take in general significantly less risks than men. This result based on a hypothetical, self-reported measure is in line with our results based on measures of actual behavior. For all variables assessing real-world financial risk taking, the difference between genders is highly significant, supporting our Hypothesis 4. In the main task, men choose the risky option 11.04 (*SD* = 3.29) times out of 25 times while women choose it 8.80 (*SD* = 3.30) times (*p* < 0.001; Cohen's *d* = 0.679). Men invest 45.77% (*SD* = 42.26) of their payoff in the final gamble while women invest only 23.92% (*SD* = 30.90). This difference is highly significant (*p* = 0.001; Cohen's *d* = 0.590). Table 3 shows the associations between risk taking in an incentive compatible task and TICS scores for men and women. Perceived chronic stress is positively and significantly associated with risk taking in the task (*r* = 0.18; *p* = 0.011), and this association holds for women (*r* = 0.29; *p* = 0.007, Table 3) and for men (*r* = 0.20; *p* = 0.035) separately as well.

Next, we test the robustness of our main finding in a regression adjusting for possible confounding factors.

### Chronic stress is associated with risk taking behavior in adults

The results of the analyses presented above indicate that, independent of gender, self-reported chronic stress is significantly correlated with financial risk taking measured in actual behavior with real stakes. We put this association to further test in seven OLS regression models (Table 5), explaining risk taking frequency in the lottery task by self-reported chronic stress, task understanding, demographic variables and psychometric variables that might interfere with chronic stress.

**Table 5:** **Regression analysis**.

	**Model (1)**	**Model (2)**	**Model (3)**	**Model (4)**	**Model (5)**	**Model (6)**	**Model (7)**
Chronic stress (TICS)	0.024^*^ 0.181^*^ (0.009)	0.030^**^ 0.228^**^ (0.009)	0.025^*^ 0.186^*^ (0.011)	0.025^*^ 0.186^*^ (0.012)	0.026^*^ 0.192^*^ (0.012)	0.024^*^ 0.183^*^ (0.012)	0.029^*^ 0.24^*^ (0.015)
Gender (0 = male, 1 = female)		−2.448^***^ −0.350^***^ (0.468)	−3.658^*^ −0.523^*^ (1.621)	−3.652^*^ −0.522^*^ (1.629)	−3.268^*^ −0.467^*^ (1.636)	−5.265^*^ −0.753^*^ (2.199)	−5.109^*^ −0.719^*^ (2.357)
Chronic stress × gender			0.014 0.191 (0.018)	0.014 0.190 (0.018)	0.013 0.172 (0.018)	0.014 0.186 (0.018)	0.010 0.131 (0.020)
Control task trials				0.050 0.004 (0.862)	0.023 0.002 (0.865)	−0.143 −0.011 (0.872)	−0.191 −0.015 (0.896)
Age					0.049 0.035 (0.097)	0.039 0.028 (0.097)	0.035 0.024 (0.104)
Income					0.768^+^ 0.128^+^ (0.409)	0.236 0.039 (0.566)	0.248 0.041 (0.589)
Economics major (0 = No, 1 = Yes)					0.548 0.077 (0.486)	0.503 0.071 (0.486)	0.419 0.058 (0.514)
Gender × income						1.112 0.284 (0.821)	1.140 0.281 (0.879)
Acute stress (VAS)							0.016 0.119 (0.010)
Anxiety (HADS)							−0.053 −0.054 (0.098)
Depression (HADS)							−0.061 −0.052 (0.105)
Medication for chronic disease (0 = no, 1 = yes)							−0.989 −0.066 (1.066)
Stress reactivity (PSRS5)							0.117 0.066 (0.144)
Constant	8.052^***^ (0.825)	8.593^***^ (0.781)	9.044^***^ (0.973)	8.892^**^ (2.777)	6.017 (3.769)	7.818^+^ (3.989)	7.362^+^ (4.167)
*R*^2^	0.033	0.153	0.156	0.156	0.180	0.188	0.208
Adjusted *R*^2^	0.028	0.145	0.143	0.138	0.149	0.153	0.149
*N*	195	195	195	195	196	195	195

The independent variable is risk-taking frequency in the task. Unstandardized, standardized coefficients (β) and robust standard errors (parentheses) are shown. Significant results have a (+) for trends where p < 0.10, with (

* ) for p < 0.05, with (

** ) for p < 0.01, and with (

*** *) for p < 0.001*.

Consistently, all seven regression models show that self-reported chronic stress explains risk taking significantly and constantly, independent of control variables. The coefficients of the TICS variables (both unstandardized and standardized) and the robust standard errors only change slightly and maintain stable.

The first model motivates risk taking by *perceived chronic stress (TICS)* for both genders. The second model details the first one with *gender* (0 = Male, 1 = Female) while the third includes the interaction between *chronic stress* × *gender*, and the fourth controls for the effect of task mis/understanding. Model five adds demographic controls: *age, income*, and *economics major (0* = *No, 1* = *Yes*), and model six looks in detail at the effect of income in the *gender* × *income* interaction. Finally, *acute stress (VAS)*, a screening for *depression (HADS-D)* and *anxiety (HADS-A), medication for chronic disease (0* = *No, 1* = *Yes*), and a brief measure of *stress reactivity (PSRS-5)* are controlled for in the seventh model.

Based on the regression models, we can confirm the robustness of the main result we presented in the previous section: chronic stress is related to risk taking. In the first model, perceived chronic stress has a positive and significant effect on risk taking. The effect slightly strengthens when gender is introduced in the second model, and the gender's coefficient shows the gender difference in risk taking. The third model encompasses an insignificant interaction between chronic stress and gender and thus shows that the difference in the effects between genders is negligible. Further, the fourth and fifth models reconfirm the robustness of the effect of chronic stress and gender on risk taking, while flagging a trend-level effect of income, which might positively influence decision-making under risk. To clarify this marginally significant effect, the sixth model includes the interaction between gender and income, which washes out the partial effect of income that arose in the previous model and shows that women are risk-cautious at all income levels. Finally, the last model reaffirms the robust effect of perceived chronic stress on risk taking, independent of stress reactivity, and excludes confounding effects from related psychological states and conditions.

In sum, the regression models confirm that self-reported chronic stress as measured by the TICS robustly explains risk taking for both genders, accounting for, but independent of, specific gender differences: chronic stress promotes risk seeking behavior in adults^12^.

## Discussion

To the best of our knowledge, we report the first study of risky decision-making in relation to perceived chronic stress and accumulated cortisol exposure. With this study, we seek to evaluate if the earlier reported effects of acute stress on risky decision perpetuate across time. We employed the TICS to measure perceived chronic stress, collected hair samples to assess accumulation of the stress hormone cortisol, and applied a standardized risk taking task, followed by self-reported risk taking and a gamble with owned money to assess decision-making under uncertainty. Our main finding is that perceived chronic stress relates significantly and robustly to incentivized risk taking behavior. We first expected, based on the research scrutinizing acute stress effects on decision-making under uncertainty (see Starcke and Brand, 2012; Buckert et al., 2014), that chronic stress will be related to risk taking behavior. While most studies on acute stress report an increase in risk taking under acute stress (for instance, Starcke et al., 2008; Lighthall et al., 2009; Pabst et al., 2013a), there are other studies that report opposite findings (Lempert et al., 2012; Delaney et al., 2014; Gathmann et al., 2014). Further, as most research on acute stress settled on the gain domain, we have only constructed trials in this domain, but we underline the fact that changing the domain, e.g., having participants make choices in the loss domain, might shift results^13^.

In the present data, both chronically stressed men and women showed increased risk taking. The link between chronic stress and risk taking was slightly stronger for women than for men, though, associations were in general relatively modest. Our finding is in line with the STARS model recently introduced by Mather and Lighthall (2012) which proposes a stress-related reward bias in decision-making, as stress triggers additional reward salience (STARS). In this sense, it might be that the riskiness of the lottery, i.e., the fact that there is a chance to gain nothing, is underestimated, while the probability to get the higher reward is overestimated. If acute stress, as shown earlier, as well as chronic stress, as shown in our data, leads to increased risk taking in humans, it might also be conceivable that a shift toward more risky behavioral decisions under stress might apply to other contexts than incentivized lotteries. However, this is highly speculative and has to be shown in future research. Nevertheless, such effects might have important consequences for individuals but also for our society, even if the observed effects are relatively modest. For example, even only slightly increased risk taking behavior by a decider in, for example, political, economic or medical decisions might have far-reaching consequences for others. Such questions are highly relevant, since the experience of chronic stress is a widespread and expanding phenomenon in our modern societies.

We were also interested in understanding whether long-term release of the stress hormone cortisol might have discernible effects on risk-taking, too. Based on the literature, we hypothesized that we might see associations between hair-sampled cortisol as a proxy for biologically measured chronic stress and risk taking. However, we did not find any correlation between accumulated cortisol exposure measured in hair samples and financial risk taking, though a positive association at trend-level between HCC and investment in the gamble surfaced for men. There are two main points worth discussing when considering our result.

First, chronicity differs between an 8-day hydrocortisone administration as in Kandasamy et al. (2014) and cortisol measured in hair samples. While the first is limited to a couple of days and pharmacologically promoted cortisol levels, the second evaluates naturally accumulated cortisol release over 1 month. Second, *ad-hoc* HCC measurements incorporate all sorts of heterogeneous factors that a standardized hydrocortisone administration might deter from: the individual's “normal” cortisol levels, his/her reactivity to stressors, his/her sensitivity or resistance to the effects of glucocorticoids, and one's own maximal reactivity in conditions of stress. While, when considering all these interindividual factors, also HCC measures might yield standard thresholds for very different persons, its averaging character might yield a different standardization than hydrocortisone dosage. If one could account for interindividual variety, pharmaceutical studies would have a better chance of determining precise effects and thresholds than *post-hoc* measurements like HCC.

One should account for the novelty of the HCC measure and, in general, for the research concerning the effects of stress, i.e., cortisol, on behavior. Just like parts of the literature surveyed, we also did not find any significant association between perceived chronic stress and HCC. Stalder and Kirschbaum (2012a) suggest retrospective bias as a potential cause. An ambulatory assessment over a longer time period or an intervention study might clarify the effect of this phenomenon. Another suggested cause is the fact that in normal populations stress exposure might be insufficiently high in order to produce physiological responses that would further render differences in HCC. We agree that academic exam stress in a student population might not be high enough to stimulate a marked chronic cortisol level. We additionally hypothesize that stress resilience and other personal characteristics like trait neuroticism might contribute to the lack of correlation between subjective measures of chronic stress and HCC. Finally, from a methodological point of view, there is recent evidence that there might be a mismatch in timing between self-reports and hair collection in studies so far: the hair collection procedure presupposes hair cutting as close as possible to the scalp and is based on the assumption that the closest 1-cm segment to the scalp encloses, on average, cortisol exposure from the most recent month, despite heterogeneity in hair growth rates. While a few millimeters are lost because of cutting instead of, for instance, shaving or plucking, terminal hair, as that on the scalp, extends a few millimeters inside the hypodermis (Wosicka and Cal, 2010). Thus, recently produced hair is uncuttable at the surface and accounts for an outgrowing lag time of 1–2 weeks (Russell et al., 2012). In sum, the cuttable hair segment at the scalp's surface might account for stress dating more than 1 month old, even if HCC levels correlate well intraindividually (Stalder et al., 2012b).

Our third hypothesis focused on gender differences. Women are known to report higher psychological distress then men (e.g., Matud, 2004; Schlotz et al., 2011) and, given that we measured chronic stress through self-reports, we expected to replicate this finding. While we did find the direction of these findings to be reliable, we could replicate them only at trend levels. The fact that women see themselves as subject to higher distress might be explained in several ways. First, given their overlapping social roles, it might indeed be the case that they are exposed to more stressors. Another plausible explanation is the fact that women might have a higher responsivity to stress, as observed for verbal responses to acute stress or heart rate responses (Kudielka et al., 2004, 2007). Finally, it might also be possible that women observe more, analyze more, and thus are more aware of their bodily and mental states (Kudielka et al., 2007).

We also replicate a gender difference in willingness to take risks in all our measures, with women being more risk averse. Interestingly, in relation to perceived chronic stress, risk seeking appears for both sexes. In what concerns the underlying motivation of this (potentially) evolutionarily-derived difference, we refrain from speculating on *post-hoc* explanations.

The present study has several limitations. First, the measures we employed are based on *ad-hoc* stress levels, without controlled chronic stress induction. Thus, our results are purely correlational and do not speak of any causal relation between chronic stress and risk taking. The same limitation applies to our results regarding the relation between perceived stress and cortisol exposure. Furthermore, our results stem from a very peculiar sample and might have thus limited generalizability: highly educated younger adults. Not least, we were suggesting above that personal characteristics could mediate stress reactivity and even the relationship between biological stress levels and perceived stress. This is one group of possible confounding variables we did not control for. In this vein, another limitation of our study is the lack of an integration of measures regarding heterogeneity in stress reactivity and sensitivity to stress. As Trautmann (2014) explains, predictions about behavior under stress become externally valid if they account for susceptibility to stress, which may, in the real world, affect economic preferences and drive self-selection into certain professions, environments and activities. Finally, the applied measure of accumulated cortisol in hair as a biological proxy for chronic stress has its limitations as discussed above. Furthermore, only a significantly reduced study sample volunteered to donate hair strands for cortisol analysis.

Nonetheless, we have opened an important avenue in investigating the effects of perceived chronic stress and accumulated cortisol exposure on behavioral decision-making. Future experimental studies should shed more light on the matter and help derive better measures for the phenomena under discussion.

## Conclusion

We explored the relation between decision-making under uncertainty and self-reported chronic stress as measured by the TICS. We additionally collected hair samples to integrate accumulated cortisol exposure as the biological facet of prolonged stress. Decision-making under uncertainty was primarily assessed through binary choices between safe and risky lotteries, and supplemented with self-reported general risk taking as well as an investment of own money in a real gamble. We discovered a significant, but modest, correlation between perceived chronic stress and actual risk taking for both genders and this positive relation is robust to multiple demographic and psychometric controls. In what regards cortisol exposure, we found no relation between general risk taking and HCC, as well as between self-reported chronic stress and HCC. However, an interesting trend-level association was observable between men's investments in the final gamble and HCC.

Our study directly contributes to the scarce research on chronic stress and decision-making. Two avenues would greatly improve the state of the knowledge in this field. First, a study performing an ecological momentary assessment of stress, resulting in more ecologically valid and retrospectively unbiased chronicity measures. Second, a higher account for interindividual variability in stress experience and reactivity, together with the account of potentially mediating effect of personal characteristics, could clarify the heterogeneity of results reported so far.

## Author contributions

All authors participated in designing the experiment, analysing the data and writing the manuscript. SC contributed most with analysing the data and running the experiment. BK contributed mostly for the physiological parts and with writing the manuscript. This justifies author order. As SC is currently on maternaty leave, CS is the corresponding author.

### Conflict of interest statement

The authors declare that the research was conducted in the absence of any commercial or financial relationships that could be construed as a potential conflict of interest.

## Footnotes

^1^Throughout this study, we refer to chronic stress as the perceived and self-reported facet of stress while to accumulated or chronic cortisol exposure as the longer-term exposure to increased cortisol secretion. However, as will be discussed later, because of a limited sample size of hair donators, we refrain from advertising, underlining, or advancing concluding statements in connection to chronic cortisol exposure and its relation to perceived chronic stress and decision-making.

^2^The average hair growth rate is 1 cm/month, so in each 1-cm segment of hair there is information about monthly exposure, with the closest cm to the scalp encompassing the latest month, the second closest the second latest month, and so on. For a thorough state-of-the-art review and detailed explanations see Stalder and Kirschbaum (2012a).

^3^See the results on the association between the two.

^4^We use the term “lottery” interchangeable with the term “gamble” and we refer to situations where outcome success is governed by chance.

^5^This was intended as a pilot for the way of displaying ambiguous choices for later studies. We will not report these data in the following.

^6^Probabilities for each possible outcome are coded by the color distribution of the bar. The height of the different colored segments signals the proportion of balls of a certain color; the proportion is also written on top of every colored segment. For instance, in the first pictogram of Figure 1 the 10/100 proportion is written on top of the yellow segment, explaining that 10 out of 100 balls in the urn are yellow.

^7^There is no significant difference in risk taking behavior or reported stress levels between class and laboratory participants (*p* = 0.17; *p* = 0.37).

^8^Inter-item reliability analysis and modified TICS descriptives are included in Tables 6, 7 in the Appendix in Supplementary Material.

^9^http://p113367.typo3server.info/index.php?id=183&L=1.

^10^We do not analyze in detail if it is exactly these ten lotteries that mostly compose risky choices, as we average behavior over the various uncertain choices.

^11^If we look at the sample that provided hair cortisol alone, the descriptive variables essentially stay the same and correlations go in the same directions, indicating that the sample is not different in any of the relevant variables.

^12^As none of the other risk measures correlates significantly with our measure of chronic stress, we do not report regressions with any of these variables.

^13^For instance, see the discrepancy between risk aversion for gains and risk seeking for losses in Kahneman and Tversky (1979).
