# Supplementary material for: Increased Risk Taking in Relation to Chronic Stress in Adults
Source: Front Psychol. 2016 Jan 29;6:2036. doi: 10.3389/fpsyg.2015.02036 (PMC4732013; doi:10.3389/fpsyg.2015.02036)

Appendix

Inter-item reliability analysis and descriptives of TICS scores

Table 6: Inter-item Reliability Analysis for the Original and Modified Versions of the TICS

Scale Label	Number of Items	Cronbach´s Alpha Original Version (Experience in the Last 3 Months)	Cronbach´s Alpha Modified Version (Experience in the Last Month)
ERDR	9	.90	.78
MANG	4	.84	.79
SORG	4	.88	.87
SOUE	6	.84	.78
SOZI	6	.88	.90
SOZS	6	.87	.85
SSCS	12	.91	.87
UEBE	8	.90	.87
UEFO	6	.87	.86
UNZU	8	.85	.81
Average	10	**.87**	**.84**
**TICS**	57	-	**.94**

Note: Cronbach´s Alpha values for the Original version are taken from the TICS manual (Schulz et al., 2004). The table rows display descriptives and analysis coefficients for the nine facets of stress comprised in nine scales (ERDR, MANG, SORG, SOUE, SOZI, SOZS, UEBE, UEFO, UNZU), a screening scale for chronic stress (SSCS), the average value of inter-item variability and the total TICS score (not existent in the original version). The nine facets of stress code the following content: pressure to be successful (ERDR), lack of social recognition (MANG), chronic anxiety (SORG), excessive social demand (SOUE), social isolation (SOZI), social tensions (SOZS), excessive workload (UEBE), mental overload at work (UEFO), and dissatisfaction at work (UNZU).

Table 7: TICS Descriptives

Sample	N	Mean	Minimum	Maximum	Median	Standard Deviation	25th Percentile	75th Percentile
Pooled	195	83.50	34	171	84.00	29.03	63.00	100.00
Females	84	87.43	37	142	85.50	24.71	70.00	105.50
Males	111	80.52	34	171	80.00	26.71	58.00	100.00

Note: The descriptives are given for the entire sample (second row), for females only (third row), and for males only (fourth row).

Further details on hair samples and hair cortisol concentrations

Table 8: Hair Cortisol Variables

	Mean	Median	SD	N (%)
Hair Cortisol Concentration (HCC) – pg/mg	7.04	5.91	3.60	51
Hair treatment	-	-	-	6 (11.76%)
Washes per week	5.12	5.00	1.68	51
Blond Hair	-	-	-	20 (39.21%)
Brown Hair	-	-	-	26 (50.98%)
Red Hair	-	-	-	1 (1.96%)
Black Hair	-	-	-	3 (5.88%)

Note: The table displays main statistics for hair cortisol- related variables.

Figure 2: Hair Cortisol and Gender

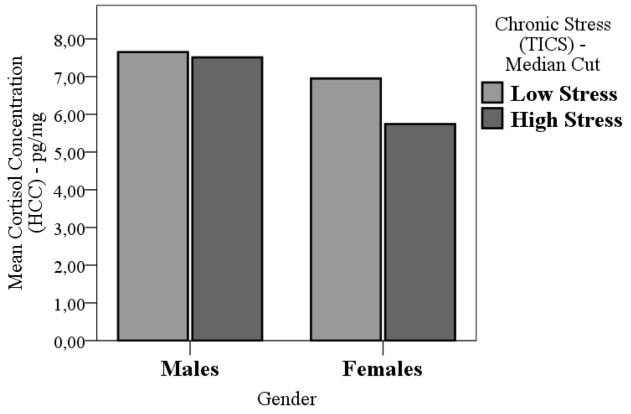

Instructions

(Please do not open the folder in front of you until you are prompted to do so!)

Dear Participant,

Thank you for being part of this study.

Before we begin the study and read the instructions, we would like to draw your attention to something you could do to further support our research. Upon completion of this experiment, you can give a small hair sample. The amount of hair we need is more or less equal to what you naturally lose in one day and we will be paying 3 euros for your time. We will examine the hair sample for hormone concentrations. Of course, the hair donation is anonymous. If you want to support us, please find us, once the study is finished, in Room 00.012, at the ground floor, in Bergheim campus. At the very end of the task folder in front of you, there is more information concerning the hair donation. You do not have to decide about this now, you can read the info sheet and ask more questions after the study today has finished.

Now we can begin the study and you are allowed to open the folder in front of you. Please do not start to look through the pages.

In front of you there is a folder containing three piles of papers. The top pile contains the task instructions that we will read together, and the instructions to create your personal identification code. The study today is completely anonymous, so from now on you will be identifying yourself only through the identification code you will create. Please, do not write your name on any of the paper sheets in the folder.

The middle pile contains the task that you will have to complete today. Please do not try to look at it before we instruct you to do so. After reading the task instructions and generating your identification code, we will continue with the task.

The last pile contains questionnaires. We ask you to fill these in after you have finished completing the task. While you are completing the questionnaires, one of the research assistants will come to you and determine your payoff from the task.

Creation of personal identification code

Prior to the beginning of the experiment, we kindly ask you to read this page carefully and follow the instructions below in order to create your personal identification code. You will be asked today, at various times points, to enter your personal identification code in order to personalize the task you are accomplishing. It is only with this identification code that we can differentiate between participants, while handling your data anonymously.

Instructions

Please answer the following questions:

	**Identification Code**
The second letter of your mother's first name: ……………………………………..	____
The day of your birthdate (for example: 03) ……………………………………..	__ __
The third letter of the street on which you currently live: ……………………………………..	____
The second and third letters of your eye color: ……………………………………..	__ __
Your height in centimeters (for example: 174) ……………………………………..	__ __ __

Example of personal identification code creation: If your mother would be called Petra, you would have been born on the 3rd of July 1954, you would now live on the Boulevard Street, your eye color would be blue and your height would be 174 cm tall; then your personal identification code would be ***E 03 U LU 174****.*

The data collected from you today is, of course, subject to confidentiality. It cannot be disclosed to any third party.

Thank you for your help!

Task Instructions

Thank you for participating in our study today. The duration of this study is approximately 40 minutes. You will receive 3 € for your participation, and will additionally receive a variable amount from the task. Details will follow. However, it is important for you to already know that you have to fill in a receipt for your payoff at the end of the study, so that we can justify proper fund usage.

We guarantee complete anonymity of your data at all times; that is, the researchers cannot connect your identity to your choices in the task or your responses to the questionnaires. You will create your own code, which serves as your identification in this study. However, for signing the payment receipt, we need your real name. Therefore, a second researcher who does not supervise your task and questionnaire completion will take care of the payment at the end of the study.

The task that you will complete today consists of 33 choices. Each choice is presented on a separate page. There are a total of 33 pages. Once you have made the decision on a certain page, please pass to the next page. You can **NOT** turn back the pages. If we observe that you turn back the pages, we must ask you to leave and you forgo your payment.

In each decision, you have to choose between two urns, graphically presented as bars. An urn always contains 100 balls of the same color (see Figure A). In the other urn there are also 100 balls, but of two

different colors (see Figure B). It may happen that you cannot see how many balls of a certain color or of another color are in the urn (see Figure C).

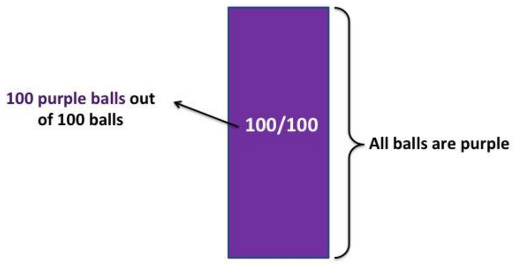

A

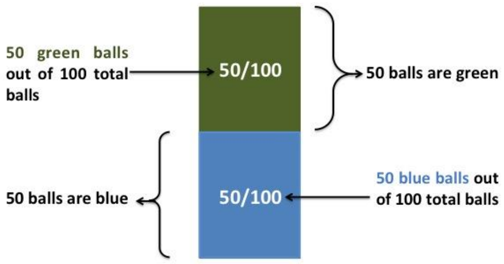

B

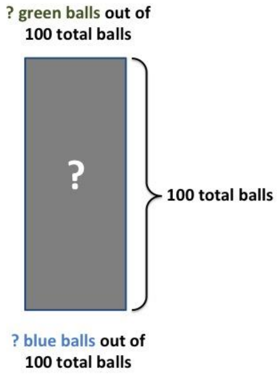

C

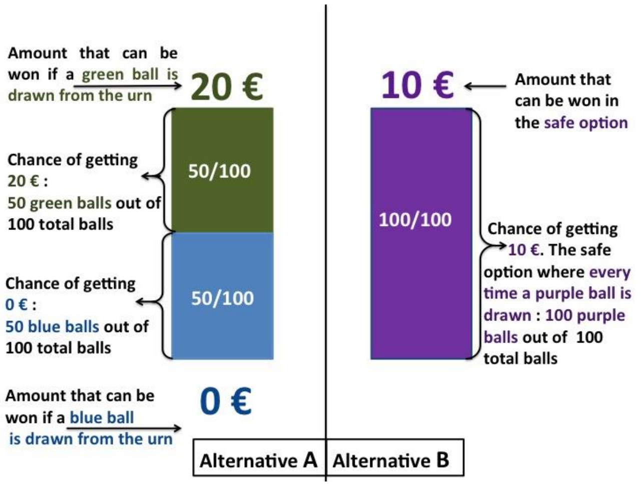

D

On each colored segment of the urn you can read the ratio of balls of that color. For example, the 50/100 ratio written over a green segment means that 50 of the 100 balls in the urn are green (see Figure D). Above and below the bar is written the amount you get when a ball of that color is drawn from the urn you selected (the amount is written in the same color as the ball it refers to).

The number and proportion of differently colored balls in the urn changes from choice to choice, as well as the amount you can gain if a certain colored ball is drawn. If you opt for the leftmost urn, circle the letter A under the leftmost urn. If you opt for the rightmost urn, circle the letter B under the rightmost urn. You need to decide on each page for one of the two urns.

You should decide on each page for the urn you truly prefer, as one of these choices you make will be played for real and paid to you at the end of the study. To decide which of the 33 choices will be selected for payment, you will draw yourself one of the 33 trial codes form a bag. You will then also determine the outcome of the lottery you chose in the respective trial by drawing a colored ball from the appropriate urn. We have prepared 33 different urns containing two types of colored balls in the same proportions as those presented in the 33 choices. As each choice contains two urns and at least one of the urns has 100 balls of the same color, we prepared bags with colored balls only for the urns that have two types of colored balls. So, these urns exist in reality and you will draw a ball from one of these urns (*urns are shown to participants*). The colors of the balls have no special meaning.

If you have any questions, please raise your hand. The research assistant will come to you and answered all your questions quietly. Of course, these instructions will remain on your desk and you can re-read them anytime you want during the study.

We now ask you not to talk to the other participants; if you try to communicate, we have to exclude you from the study.

Please start by creating your personal identification code and then completing the choices in the task.

Real urns that enabled real randomness

1. 33 trial codes are put in a bag; the participant blindly draws one trial code showing what urn will be chosen further.

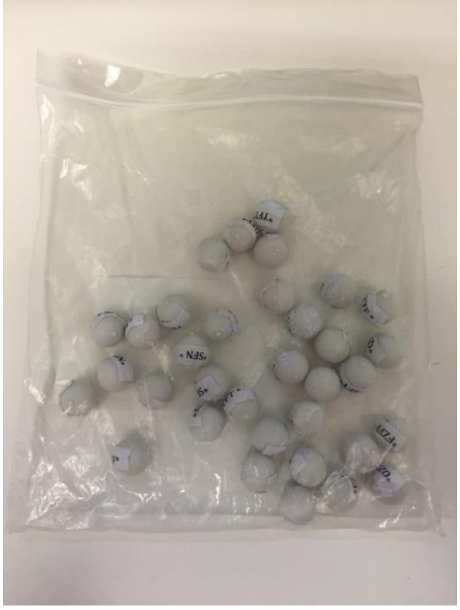

If the risky/ ambiguous urn was chosen in the respective trial, the participant blindly draws a ball from the appropriate urn and his/ her final payment is thus established.

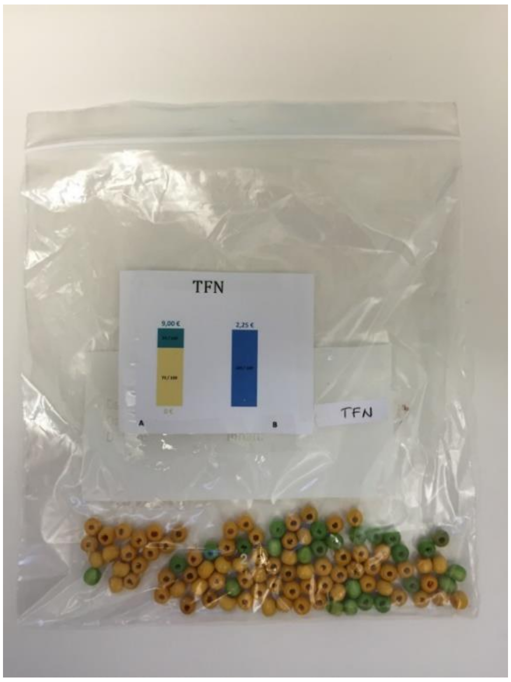

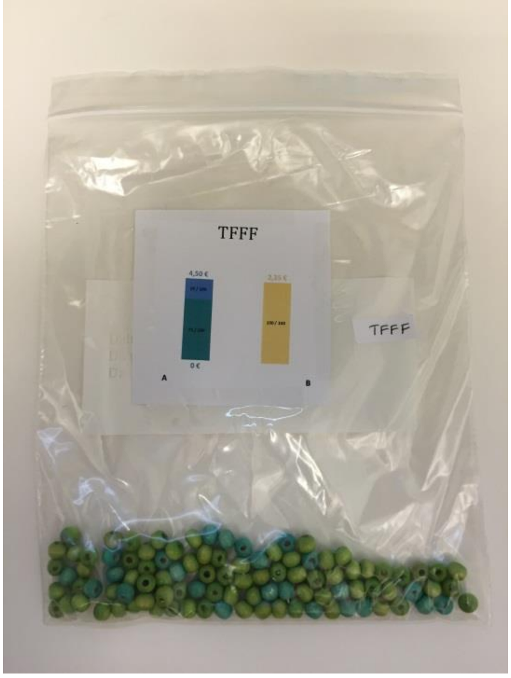

Supplement: Supplementary file 1 [file DataSheet1.pdf]
